# Randomized Controlled Trials on COVID-19 Should Be Accurate and Trustworthy

**DOI:** 10.4269/ajtmh.21-0836a

**Published:** 2021-08-30

**Authors:** Ben W. Mol

**Affiliations:** Department of Obstetrics and Gynaecology Monash Medical Centre 246 Clayton Rd. Clayton, Victoria 3168 Australia E-mail: ben.mol@monash.edu

Dear Sir,

Abd-Elsalam et al.^1^ recently published a randomized clinical trial on the effectiveness of hydroxychloroquine in patients with COVID-19. I read their article with interest and came across some inconsistencies in the data provided.

In particular, I have the following concerns:

1. The timelines in the study seem not feasible. The study states that participants in the study were “admitted between March and June 2020,” and then states, “All the patients were followed up for 4 weeks.” The article was received by the Journal on July 17, 2020. This raises the question of how follow-up could have been completed by mid-July, with all data analyzed.
2. The study recruited similar patients in the same period in the same centers for two other studies.^2^^,^^3^ Could the authors explain how it was decided to which study a patient was recruited?
3. According the trial registry (NCT04353336), the study was set to continue until September 23, 2030. The trial registry also specifies the initial sample size as 40 (NCT04353336), which appears to have been changed to 200 after completion of the recruitment. The article does not provide a prospective sample size calculation.
4. Would the authors be able to provide an explanation on how they calculated the 15 *P* values in Table 2? My own calculations led to considerably different results.
5. Some of the study data appear improbable. There are nine instances of a *P* value between 0.05 and 0.10, four with a *P* value of 0.07 in Table 2. Multiple variables have similar decimals (i.e., 0.26 and 28.17), and there are widely different SDs between groups (i.e., 13.45 versus 179.2). Might some values have been duplicated in error?
6. Would the authors be able to explain the 20% difference in recovery rate between study arms, without any difference in “duration to clinical improvement” or “duration to hospital discharge” (Table 3)?

I encourage the authors to share their original data and to provide clarification, based on the concerns listed here, to support the remarkable results of this study.
